# Correlation of Vernalization Loci *VRN-H1* and *VRN-H2* and Growth Habit in Barley Germplasm

**DOI:** 10.1155/2013/924043

**Published:** 2013-04-02

**Authors:** Mohsen Mohammadi, Davoud Torkamaneh, Hamid-Reza Nikkhah

**Affiliations:** Cereals Research Department, Seed and Plant Improvement Institute (SPII), P.O. Box 4119, Karaj, Iran

## Abstract

Vernalization requirement is a key component in determining the overall fitness of developmental patterns of barley to its environment. We have used previously reported markers and spring-sown growth habit nursery to characterize the genotypes of barley germplasm in an applied barley breeding ground to establish a baseline of information required to understand the relationship between adaptation of autumn-sown barley germplasm in diverse regions with warm (W), moderate (M), or cold climates (C). This study revealed that twenty entries were detected with the presence of the vernalization critical region in *VRN-H1* locus and complete presence of the three geneclusters *ZCCT-Ha*, *-Hb*, and *-Hc* in *VRN-H2* locus represented as genotype *vrn-H1/Vrn-H2 (V1w/V2w)*. Of these genotypes, 17 entries showed winter growth habit whereas the remaining three revealed facultative growth habit indicating reduced vernalization requirements possibly due to *VRN-H3* and photoperiod sensitivity loci as compared to the landmark winter growth habit entries in this group. Twenty-four entries were detected with the lack of vernalization critical region in *VRN-H1* locus but complete presence of the three geneclusters *ZCCT-Ha, -Hb*, and *-Hc* in *VRN-H2* locus represented as genotype *Vrn-H1/Vrn-H2 (V1s/V2w)*. However, only half of these germplasms were identified with spring growth habit in spring-sown nursery, and the rest of the germplasms in this group revealed facultative growth habits due to possible variation in the length of deletion in *VRN-H1*. Four germplasms showed vernalization insensitive phenotype due to the lack of a functional *ZCCT-Ha* and/or *ZCCT-Hb* alleles in *VRN-H2* and the deletion in the vernalization critical region of *VRN-H1*. These germplasms revealed acomplete spring type growth habit. Only one entry showed reduced vernalization requirement solely due to the deletion in functional *ZCCT-Hb* allele in *VRN-H2* and not due to the deletion in the vernalization critical region of *VRN-H1*.

## 1. Introduction

Increases in yield and adaptation of cereals rest on life cycle adjustments to environmental constraints explained, in part, by the transition from vegetative to reproductive growth, flowering, and maturity [1, 2]. Optimal fitness of developmental patterns to the environment takes place when the crop utilizes most of inputs from the environment on the one hand and on the other hand escapes from deleterious effects of the adverse environmental conditions (i.e., frost stress and late maturity) [3], [4] leading to superior crop performance and yield potential [3]. Barley is a temperate cereal grown in diverse climatic regions from spring growth habit to winter growth habit [5]. Some barley varieties require prolonged exposure to cold temperatures to initiate flowering, a process referred to as vernalization, whereas others initiate flowering without any obligation for vernalization. Therefore, learning more about genetic variation of loci governing vernalization in barley breeding materials is beneficial for developing new cultivars for target environments. 

The concept of vernalization sensitivity in barley is different from that in wheat and the sensitivity to low temperature exposure in barley is not an absolute concept. For a vernalization-sensitive wheat, genotype to induce flowering, the seedlings require exposure to low temperatures (4–6°C) for 4–6 weeks [6]. The vernalization-sensitive wheat plant does not flower if the vernalization requirement is not fulfilled, whereas an unvernalized vernalization-sensitive barley genotype may induce flowering in late cropping season which is not efficient economically [7]. A three-locus epistatic model of vernalization sensitivity has been proposed by Takahashi and Yasuda [8], which then was reduced to two-locus epistatic model on the ground that allelic variations in *VRN-H3* locus is limited [9]. According to this model, vernalization response in barley is mainly determined by *VRN-H1* locus on chromosome 5H and *VRN-H2* locus on chromosome 4H, where genotypes with dominant allele *Vrn-H2* on *VRN-H2* locus and recessive allele *vrn-H1* on *VRN-H1* locus require the greatest vernalization requirement. 

The candidate gene responsible for the *VRN-1* locus in wheat and barley is a MADS-box floral meristem identity gene (HvBM5A). High expression of *VRN1* is required for the initiation of reproductive development. In some varieties of barley and wheat, MADS-box gene is expressed at high basal levels without requiring a prolonged exposure to cold temperature. Whereas other varieties require prolonged exposure to cold temperature to induce expression of MADS-box at higher levels of expressions required for transition to reproductive stages [10–13]. Further genetic studies revealed that mutations in the promoter or deletions within the first intron of HvBM5A gene lead to dominant Vrn-H1 spring type alleles (nonvernalization requiring) and are known to be responsible for the high level of expression of HvBM5A and the reductions of vernalization requirements in barley [10, 13, 14]. Investigation of a large collection of barley germplasm narrowed down the allelic variations of *VRN-H1* locus to nine haplotypes (1A, 1B, 2, 3, 4A, 4B, 5A, 5B, and 5C) within the European germplasm [1, 2] of which haplotypes 1A and 5C are typical winter type alleles. The distinction between winter and spring *Vrn-H1* haplotypes is based on the presence or absence of the key regulatory element localized in the first intron of barley, a so-called “critical region” [14, 15]. Further investigation of variation in *Vrn-H1* winter haplotypes revealed a 486-bp discriminative deletion found only within the solo long terminal repeat (LTR) of haplotype 5C but not on 1A resulting in amplification of 344 bp and 830 bp products in winter haplotypes 5C and 1A, respectively [16]. 

The *VRN-H2* locus is a dominant repressor of flowering. Genotypes lack a dominant (functional) copy of *VRN2* flower early [17], The candidate gene proposed for Vrn-2 locus in wheat and barley is a *ZCCT* zinc finger CCT domain transcription factor [17], Transgenic and RNA interference studies have shown that the *ZCCT* gene was responsible for the variation in the vernalization requirement in wheat [17] Loss-of-function or complete deletion mutation in the coding region of this gene leads to recessive spring type vrn-H2 growth habit alleles. The *ZCCT* candidate gene cluster in *VRN-H2* locus consists of three tightly linked genes, *ZCCT-Ha*, *ZCCT-Hb*, and *ZCCT-Hc* [18], and more references. Laurie et al. [19] have mapped *VRN-H2* locus on the distal part of as mentioned by long chromosome 4H in barley. *VRN-H3*, the third vernalization locus in barley, is located on chromosome 1H Takahashi and Yasuda [8]. The candidate gene proposed for this locus is *HvFT1* which is homologous to the *Arabidopsis FT* gene [20, 21]. The elevated expression of *HvFT1* accelerates flower development [20]. Dominant *Vrn-H3* allele results in elevated expression levels of *HvFT1*. In genotypes requiring prolonged exposure to low-temperature treatment for flower development dominant *Vrn-H3* allele provides a bypass of the vernalization requirement [20]. The expression of *VRN-H3* is also mediated by a pathway downstream of *VRN-H1* and *VRN-H2* and interactions with alleles at *VRN-H2* and *PPD-H1* loci. When a genotype possesses recessive *vrn-H2* or vernalization fulfillment represses dominant *Vrn-H2*, *HvFT1* is induced under long-day conditions and accelerates flower development, though this pathway requires active *Ppd*-*H1* allele [22–24].

The barley germplasms grown in Iran are either developed within the country or they were introduced from the ICARDA barley breeding program. The knowledge of germplasm growth habit is limited to flowering in spring sown nurseries. Neither the allelic composition and distribution of vernalization sensitivity loci have been characterized, nor it is clear as to how such allelic variations contribute to the overall adaptation of the country's barley germplasm through the adjustment of flowering time. In this study, we unravel allelic distribution of vernalization loci in barley. The objective of this investigation is the identification of the allelic combinations of *VRN-H1* and *VRN-H2* loci deployed in a collection of contemporary cultivars by using allele-specific molecular markers and to enhance our understanding of the underlying factors governing overall adaptation of barley to various geographical regions within the barley breeding program. 

## 2. Materials and Methods

### 2.1. Plant Materials

 A total of 51 barley genotypes including cultivars that either developed locally or introduced from external breeding programs, elite experimental lines, and local landraces collected from Iran were selected. The climatic diversity represents regions with moderate temperatures, areas with terminal heat stress, and regions prone to frost stress. In some regions with either moderate temperature or terminal heat stress, the barley production is also under drought and salinity conditions. 

### 2.2. Growth Habit Phenotyping

 The growth habit nursery was planted on a single row of one-meter-long plot for each entry during spring 2012 where the minimum temperature did not fall below. Growth habit phenotypes were scored in three categories of W, F, and S representing genotypes with no tiller flowered on the row, some tillers flowered, and all tillers fully flowered, respectively. Genotypes were scored as spring when all tillers of the entire row flowered, as facultative when only few tillers were flowered, and winter when none of the tillers on the entire row flowered by the end of summer 2012.

### 2.3. DNA Extraction

 Small-scale nucleic acid isolation method suitable for marker-assisted selection procedures was used to extract DNA from young leaves of barley genotypes. Fresh leaf tissue of two-week old barley plants were flash frozen in liquid nitrogen and ground into a fine powder. Extraction was performed in 2 mL sterile tubes where a quarter of each 2 mL tube was filled with frozen leaf powder. Then, 0.9 mL of preheated (up to 65°C) CTAB buffer (2% (w/v) cetyltrimethylammonium bromide, 200 mM Tris/HCl pH 8.0, 20 mM EDTA pH 8.0, 1.4 M NaCl, and 1% (v/v) freshly added *β*-mercaptoethanol) was added to each tube. Extraction mixes were incubated for 45 min at 65°C. Precooled chloroform: isoamyl-alcohol mixture (24 : 1) (0.9 mL) was then added to the heated tubes. The extraction mixes were then centrifuged at max speed (13600 xg) for 15 minutes at 4°C. The upper layer was then transferred into a new tube and supplemented with 0.7 volumes of precooled (−20°C) isopropanol and 0.1 volumes of 3 M ammonium acetate. DNA was allowed to precipitate at −20°C for 45 minutes. The precipitates were pelleted by centrifugation at max speed for 10 minutes at 4°C. The pellets were washed at least once with 300 *μ*L of precooled 70% (v/v) ethanol. DNA was finally dissolved in 70 *μ*L of nuclease free water. 

### 2.4. DNA Amplifications and Visualization

Deletion-specific primers HvBM5.84F and HvBM5.85R [25] were used to discriminate between recessive and dominant alleles at *VRN-H1* locus based on the presence or absence of the regulatory element in intron I of *HvBM5*. Thermal cycling profile for these primers consisted of an initial denaturing step of 94°C for 3 min, followed by 35 cycles of denaturation at 94°C for 35 s, annealing at 58°C for 45 s, and extension at 72°C for 45 s. The PCR reaction was terminated by a final extension of 72°C for 10 min. The discrimination between two winter haplotypes was assessed by using primers HvBM5A-intronI-F3b and HvBM5A-intronI-R3b after Cockram et al., [16]. PCR was initiated at 95°C for 3 min, followed by 35 cycles of denaturation at 94°C for 1 min, annealing at 54°C for 45 s, and extension at 72°C for 80 s. The PCR reaction was terminated by a final extension of 72°C for 5 min. Three deletion-specific primer pairs were used to assess the deletions in three-gene cluster *VRN-H2* locus that is, *ZCCT-Ha*, *ZCCT-Hb*, and *ZCCT-Hc* [20, 25]. The amplification of a physically linked locus HvSNF2 was also used as a positive control as described by Karsai et al., 2005. Thermal cycling profile for these primers consisted of an initial denaturing step of 94°C for 4 min, followed by 35 cycles of denaturation at 94°C for 45 s, annealing at 54°C for 45 s, and extension at 72°C for 1 min. The PCR reaction was terminated by a final extension of 72°C for 10 min. Full list of primer names and sequences are listed in Table 1. PCR reactions of 25 *μ*L contained 1 *μ*L DNA, 2.5 *μ*L 10X Taq buffer, 1.5 mM MgCl_2_, 1 *μ*L dNTP mix (2.5 mM of each dNTP), 0.4 pmol of each primer, and 1 unit of Taq polymerase. PCR reactions were performed in an Eppendorf Mastercycler (Eppendorf AG, Hamburg, Germany) or T100 Cycler (Bio-Rad Laboratories, Inc., USA). DNA amplifications were visualized, after ethidium bromide staining and separation on 1% or 1.5% agarose gel electrophoresis by using UVItec Gel Document system (UVItec Limited, Cambridge, UK).

## 3. Results and Discussion

**Figure 1:**
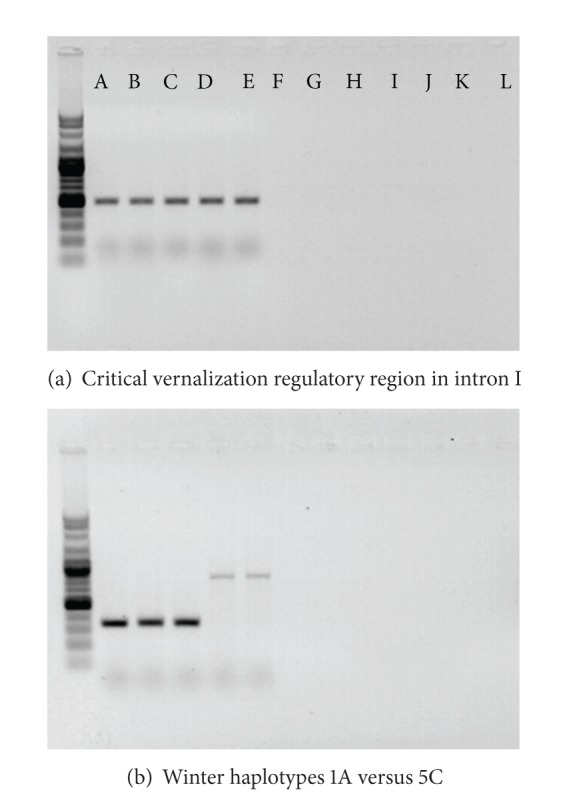
(a) Amplification of 437 bp PCR product, by using primers HvBM5.84F and HvBM5.85R, indicative of presence of critical vernalization regulatory region in *VRN-H1* locus—a winter type recessive *vrn-H1* allele in A: Karoon, B: Shahr-e-Kord, C: Arigashar, D: Bahman, and E: Radical, and no amplification suggestive of a deletion in critical vernalization region in *VRN-H1* locus leading to a spring type dominant *Vrn-H1* allele in F: Torkman, G: Jonoob, H: Kavir, I: Nosrat, J: Nimrooz, K: Yousof, and L: Morex. (b) Discrimination between winter haplotypes 5C and 1A shown by amplification of 344 bp PCR band, by using primers HvBM5A-intronI-F3b and HvBM5A-intronI-R3b, in A: Karoon, B: Shahr-e-Kord, and C: Arigashar and amplification of 830 bp PCR band by using the same primers in D: Bahman and E: Radical.

We used allele-specific primers to identify allelic variations of vernalization loci *VRN-H1* and *VRN-H2* in barley germplasm. We have also evaluated flowering potentials of the germplasm in the spring-sown nursery to establish whether or not they require vernalization for flowering. Three cultivars were used as positive controls in this study. They included cultivars “Nure” and “Igri” with genotype *vrn-H1/Vrn-H2* (*V1w/V2w*) as typical winter growth habit and cultivar “Morex” with genotype *Vrn-H1/vrn-H2 *(*V1s/V2w*) as a typical spring growth habit [14].  Table 2 represents the relationship between the allelic combinations of *VRN-H1* and *VRN-H2* and the predicted growth habit. A simplified notation for spring type versus winter type alleles is also provided to avoid confusion of alleles. PCR amplification using primers HvBM5.84F and HvBM5.85R resulted in PCR products of 437 bp in 21 entries with the same size as observed for Nure and Igri, indicating the presence of the vernalization critical region in intron I region of *VRN-H1* locus suggestive of winter type recessive *vrn-H1* allele (Table 3). Further assessment of these genotypes by means of primer pairs HvBM5A-intronI-F3b and HvBM5A-intronI-R3b resulted in PCR products of 830 bp and 344 bp indicative of winter haplotypes 1A and 5C, respectively (Table 2). Representative gel pictures showing amplifications of 437 bp (vernalization critical region) and discrimination of winter haplotypes 1A versus 5C are shown in Figure 1, respectively. Other genotypes (*n* = 30) did not yield any PCR products when amplified by primers HvBM5.84F and HvBM5.85R suggestive of a deletion in Vrn-H1 leading to a spring type dominant *Vrn-H1* allele (Table 3). 

**Figure 2:**
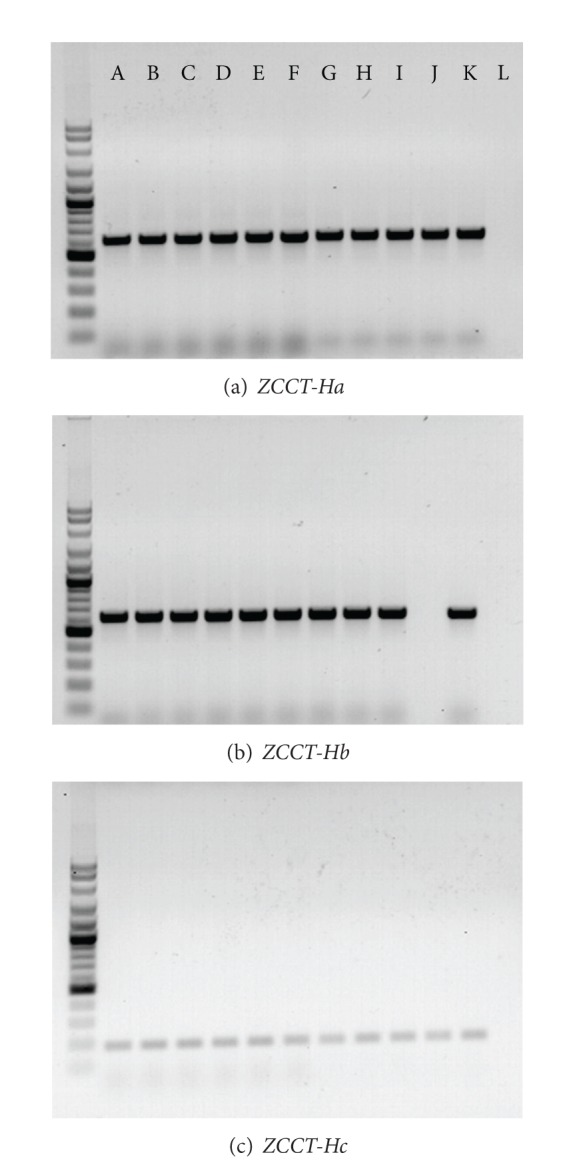
(a) Amplification of 600 bp PCR product, by using primers ZCCTH.14F and ZCCTH.19R, indicative of presence of *ZCCT-Ha* in all germplasms tested except L: Morex. (b) Amplification of 600 bp PCR product, by using primers ZCCTb.8F and ZCCTb.11R, indicative of the presence of *ZCCT-Hb* in all germplasms tested except J: Nimrooz and L: Morex (c) Amplification of 200 bp PCR product, by using primers ZCCT.HcF and ZCCT.HcR, indicative of the presence of *ZCCT-Hc* in all germplasms tested except L: Morex. Germplasms included in this figure include A: Karoon, B: Shahr-e-Kord, C: Arigashar, D: Bahman, E: Radical, F: Torkman, G: Jonoob, H: Kavir, I: Nosrat, J: Nimrooz, K: Yousof, and L: Morex.

PCR amplification using primers HvSNF2.01F and HvSNF2.04R as positive control primers for Vrn-H2 locus resulted in amplification of a PCR band sized 500–700 bp in all genotypes invariably. When assessed by three *ZCCT* allele-specific primer pairs, only the positive control “Morex” lacked all three *ZCCT* genes. Most of the genotypes were characterized as winter type dominant *Vrn-H2* allele because they yielded the PCR products of coding regions of *ZCCT-Ha* (600 bp), *ZCCT-Hb* (600 bp), and *ZCCT-Hc* (200 bp) (Figure 2). In five genotypes, that is, 1-BC-80318, 1-BC-80021, Tokak, ND-82-10, and Nimrooz, the gene *ZCCT-Hb* was found deleted. Four of the genotypes were characterized by a spring type dominant *Vrn-H1* allele and only 1-BC-80318 possessed the full length winter type recessive *vrn-H1* allele. In genotype Bereke.54 both *ZCCT-Ha* and *ZCCT-Hb* were absent, and it showed a spring type dominant *Vrn-H1* allele (Table 3). Based on a single variety “Fan” observation, Dubcovsky et al. [18] provided preliminary evidence that, of the three tightly linked *ZCCT* genes in barley, *ZCCT-Hb* is not sufficiently deterministic for winter growth habit. One year later, when examining the effect of daylength on expression of *VRN-H1* and *VRN-H2*, Trevaskis et al. [22] discovered that in a vernalization requiring winter barley the expression of *VRN-H1* is not regulated by daylength. In contrast, daylength was shown to reduce the expression of *ZCCT-Ha* and *ZCCT-Hb* but not *ZCCT-Hc*. This may indicate that both *ZCCT-Ha* and *ZCCT-Hb* may be sufficiently deterministic for winter growth habit, and deletion of either of the genes may result in the reduced vernalization requirement. Therefore, for the representation of our results, we have decided to denote recessive *vrn-H2* where *ZCCT-Hb* was absent on the basis of the fact that this may reduce deterministic effect of *Vrn-H2* locus for a vernalization requirement (Table 3). 

In total, 20 genotypes were identified as *vrn-H1/Vrn-H2* (*V1w/V2w*)—a typical genotype for winter growth habit. Almost all entries in this group revealed winter growth habit phenotype in our growth habit nursery except for 1-BC-80392, 1-BC-80207, and 1-BC-80392 that showed facultative growth habit indicating reduced vernalization requirements as compared with others in this group. In this group, a majority of cultivars grouped with landmarks of winter barley genotypes in our study, that is, “Nure” and “Igri” where the *VRN-H1* locus harbored the winter haplotype 1A. Cultivars “Bahman,” “Goharjo,” and “Makoee” are well-adapted winter barley varieties grown in zone C with acceptable levels of winter survival. In contrast, cultivars Karoon and landrace accessions Arigashar and Ashar from Baluchestan Province revealed genotype of *vrn-H1/Vrn-H2* (*V1w/V2w*) where the *VRN-H1* locus harbored the winter haplotype 5C. 

A total of 24 genotypes were identified as *Vrn-H1/Vrn-H2* (*V1s/V2w*) with expected spring growth habit. These genotypes were grouped with landmarks of spring growth habit barley cultivars in our study, that is, “Yousof” and “Kavir.” This group is characterized with a deletion of the vernalization critical region from *VRN-H1* intron I. However, in this study we do not report the length of deletion on spring type dominant *Vrn-H1* alleles. Therefore, some of these genotypes might represent minimal or reduced vernalization requirements. Various studies have reported that sole deletion observed in the vernalization critical region in *VRN-H1* locus may not be sufficient for the removal of the vernalization requirement. Rather, the length of intron I associated with the deletion is more relevant to the reduction in vernalization requirements [27, 28]. Our phenotyping growth habit data revealed that only half of these entries fully flowered in growth habit nursery, and others showed degrees of facultative growth habit. We concluded that there may be variation in length of deletion in *VRN-H1* locus amongst genotypes in our study, and therefore, some of these genotypes represent reduced vernalization requirements. 

Four germplasms showed vernalization insensitive phenotype due to the lack of a functional *ZCCT-Ha* and/or *ZCCT-Hb* alleles in *VRN-H2* and the deletion in the vernalization critical region of *VRN-H1*. These germplasms were Nimrooz, ND-82-10, Tokak, and Bereke-54 which together with Morex revealed complete spring type growth habit. The only entry with the reduced vernalization requirement solely due to the deletion in functional *ZCCT-Hb* allele in *VRN-H2* locus and not due to the deletion of the vernalization critical region was “1-BC-80318”. This entry revealed a facultative phenotype in the growth habit nursery.

**Figure 3:**
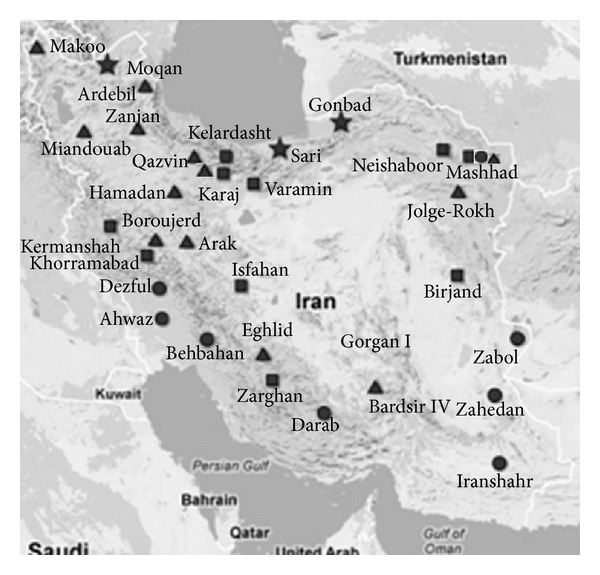
Geographical regions based on the current climatic classification for the barley breeding program. Agricultural areas located in zone W-N (star) are characterized by warm and humid climate during the reproductive stage. Areas located in zone W-S (circle) have hot summers and are prone to heat stress and drought. Zone M (square) does not have an incidence of extreme temperatures. Regions located in zone C (triangle) are characterized by prolonged freezing temperatures and frequent frost damage. The map was generated by using GPS Visualizer server available at (http://www.gpsvisualizer.com/), and the names of locations were added manually in power point. This figure is shown best at this size or smaller in the printed version.

The barley breeding program in Iran operates on the basis of viewing the country as four mutually distinct climatic characteristics (Figure 3) on the basis of variation in temperature and humidity. This environmental classification has been in place for nearly three decades and includes W-N, W-S, M, and C. The region W-S, characterized by warm and humid climate particularly in reproductive growth stages, is mostly distributed in the north of the country. The regions classified as W-S are mainly in southern Iran and encompass regions with rapidly increasing temperatures in the spring followed by a very hot summer leading to a combined heat and likely drought stress. In region M, the growing season is normally not associated with an extreme temperature or humidity conditions. This region is mostly distributed in the central, western, eastern, and northeastern parts of the country. Regions in zone C are prone to frost damage during the vegetative stage (C), and therefore, winter survival plays a vital role in crop yield. 

In Iran, barley is predominantly sown in autumn each year and harvested in the summer of the following year. Spring-sown barley production is not practiced in Iran. What is neglected from this mutual classification of regions—with respect to vernalization requirements—is that nearly all cultivated regions of the country including W-N, W-S, and M are entailed to a mild winter which yet is sufficient for fulfilling vernalization requirements. That explains why some of the cultivars developed or introduced for regions W (Karoon and Jonoob) and M (Nosrat and Rihane) are entailed with full or reduced vernalization requirement. This study drew a baseline understanding of the relationship between genotypes at vernalization loci in barley and growth habit, expected flowering time, and winter survival as related to various geographical regions in an applied barley breeding ground. Genotypic analysis of barley germplasm as represented here increased our awareness of allelic variations in vernalization loci and the adaptation of barley germplasm to various regions of the country. Such information may be used in breeding practices for designing crosses for recombining allelic forms of *VRN* in barley and attempting marker-assisted selection for earliness and winter survival. However, further investigation is required to advance our knowledge about SNP variation and InDel evens in *VRN-H3* locus and allelic variation in barley photoperiod response loci, that is, *PPD-H1* and *PPD-H2*. 

## Figures and Tables

**Table 1:** The list of primers names, sequences, and expected PCR product bands used in this study with the original names obtained in the references cited.

Vrn-H1			
HvBM5.84F	5′-TGAGGGTATGAGTGGCGCTAG-3′	437 bp ±	Koti et al., 2006 [25]
HvBM5.85R	5′-TCTCATAGGTTCTAGACAAAGCATAG-3′		
HvBM5A-intronI-F3b	5′-CTTGCATGTGTTGTCGGTCT-3′	344/830 bp	Cockram et al., 2009 [16]
HvBM5A-intronI-R3b	5′-GCTGGGACAAGACTCTACGG-3′		
Vrn-H2			
ZCCTH. 14F:	5′-CAAGGAATATCAAGTACATATCTGC-3′	600 bp ±	Szűcs et al., 2007 [26]
ZCCTH. 19R:	5′-CCGTATTTATTGAGTTGGTGGTG-3′		
ZCCTb. 8F:	5′-GCATCAATGCACCCTACCTCTT-3′	600 bp ±	Szűcs et al., 2007 [26]
ZCCTb. 11R:	5′-GGAAAACAATGGTGAGAGTAGTACAG-3′		
ZCCT. HcF:	5′-CACCATCGCATGATGCAC-3′	200 bp ±	Yan et al., 2006 [20]
ZCCT. HcR:	5′-TCATATGGCGAAGCTGGAG-3′		
HvSNF2.01F	5′-CCTGAAGCGAGTATCCATATGC-3′	500–700 bp	von Zitzewitz et al., 2005 [14]
HvSNF2.04R	5′-GCTGCATTATAGAGAAACAACAACG-3′		

**Table 2:** The predicted growth habit for each genotypic class is listed based on allelic combinations of two-locus *VRN-H1/VRN-H2* flowering model. A simplified notation for allelic combinations in relation to growth habit is also provided.

Allelic combinations	Simplified notation	Predicted growth habit
*vrn-H1/Vrn-H2 *	*V1w/V2w *	Winter
*vrn-H1/vrn-H2 *	*V1w/V2s *	Facultative
*Vrn-H1/Vrn-H2 *	*V1s/V2w *	Spring
*Vrn-H1/vrn-H2 *	*V1s/V2s *	Spring

**Table 3:** Germplasm evaluated for allelic variations of *VRN-H1* and *VRN-H2* loci along with their primary cultivation area predicted and observed growth habits are presented. C, M, W-S, and W-N represent cold, moderate, warm-south, and warm-north cultivation regions, respectively. W, F, and S represent winter, facultative, and spring growth habit, respectively.

Genotype/germplasm	Row type	Pedigree	Regions cultivated	VRN-H1 (437 bp)	5C versus 1A	HvSNF	VRN-H2 ZCCT-Ha	VRN-H2 ZCC-Hb	VRN-H2 ZCCT-Hc	Predicted GH	Observed GH
*vrn-H1/Vrn-H2 (V1w/V2w) *											

Goharjo	6	Landrace	C	+	830 (1A)	+	+	+	+	W	W
Michailo		Michailo		+	830 (1A)	+	+	+	+	W	W
Radical	6	Radical	C (CB, ICARDA)	+	830 (1A)	+	+	+	+	W	W
Makoee	6	Star/FAO	C	+	830 (1A)	+	+	+	+	W	W
Bahman	6	WA 2196-68/NY6005-18, F1//Scotia I	C	+	830 (1A)	+	+	+	+	W	W
Dasht	2	Probestdwarf (France)	W	+	830 (1A)	+	+	+	+	W	W
L1242	6	L1242	M, C	+	830 (1A)	+	+	+	+	W	W
Karoon	6	Strain205	W-S	+	344 (5C)	+	+	+	+	W	W
Nure	2	Fior 40/Alpha2//Baraka	C	+	830 (1A)	+	+	+	+	W	W
Igri	2	820/1427//Ingrid	C	+	830 (1A)	+	+	+	+	W	W
1-BC-80392	6	Landrace	M, C	+	830 (1A)	+	+	+	+	W	F
1-BC-80453	6	Landrace	M, C	+	830 (1A)	+	+	+	+	W	W
1-BC-80458	6	Landrace	M, C	+	830 (1A)	+	+	+	+	W	W
1-BC-80395	6	Landrace	M, C	+	344 (5C)	+	+	+	+	W	W
Shahr-e-kord	6	Landrace		+	344 (5C)	+	+	+	+	W	W
Arigashar	6	Landrace	W	+	344 (5C)	+	+	+	+	W	W
Ashar	6	Landrace	W	+	344 (5C)	+	+	+	+	W	W
1-BC-80087	6	Landrace	M, C	+	344 (5C)	+	+	+	+	W	W
1-BC-80207	6	Landrace	M, C	+	344 (5C)	+	+	+	+	W	F
1-BC-80017	6	Landrace	M, C	+	344 (5C)	+	+	+	+	W	F

*vrn-H1/vrn-H2 (V1w/V2s) *											

1-BC-80318	6	Landrace	M, C	+	344 (5C)	+	+	−	+	F	F

*Vrn-H1/Vrn-H2 (V1s/V2w) *											

MB-87-10	6	82S:510/3/Arinar/Aths//DS 29	M	−	NA	+	+	+	+	S	S
Nosrat	6	Karoon/Kavir	M	−	NA	+	+	+	+	S	F
Rihane	6	Rihane	M	−	NA	+	+	+	+	S	F
MBS-87-12	6	Roho/Mazorka//Trompilo	M	−	NA	+	+	+	+	S	F
Valfajr	6	CI-108985/Egypt	M, C	−	NA	+	+	+	+	S	F
Torkaman	6	Rihane04/FAO	W-N	−	NA	+	+	+	+	S	F
Gorgan4	2	Herta/Sweden	W-N	−	NA	+	+	+	+	S	S
Jonob	6	Gloria “s”/ Copal “s”/CIMMYT	W-S	−	NA	+	+	+	+	S	F
Aras	2	Arumir/Europe	W-N	−	NA	+	+	+	+	S	S
Yosef	6	L.527/Chn-01//Gostoe/4 /Rhn-08/3/Deir Alla106// DL71/Strain205	M, W	−	NA	+	+	+	+	S	S
20269	6	Kavir/Badia	M	−	NA	+	+	+	+	S	F
20371	6	Legia	C, M	−	NA	+	+	+	+	S	S
1-BC-80162	6	Landrace	M	−	NA	+	+	+	+	S	S
1-BC-80628	6	Landrace	M	−	NA	+	+	+	+	S	S
Nik	6	Lignee 527/NK1272//JLB 70-63 MB-83-14	M	−	NA	+	+	+	+	S	S
MB-82-12	2	Novosadski-444	M	−	NA	+	+	+	+	S	F
EHD-85-8	6	CIRU/3/AGAVE/SUM BARD400//MARCO/4/ PETUNIA 1	M	−	NA	+	+	+	+	S	S
Kavir	6	Arivat (Atlas/Vaughn)	M	−	NA	+	+	+	+	S	S
Fajr30	6	Lignee131/Gerbel//Alger-Ceres/3/Jonoob	M	−	NA	+	+	+	+	S	F
Teser.93		Teser.93		−	NA	+	+	+	+	S	S
Sararood	2	Landrace	M	−	NA	+	+	+	+	S	F
Badia (20261)	6	Badia	M	−	NA	+	+	+	+	S	S
EH-83-7	6	Congona/Borr	M	−	NA	+	+	+	+	S	S
MBS-82-5		Salt tolerant (5 shoori)	M	−	NA	+	+	+	+	S	F

*Vrn-H1/vrn-H2 (V1s/V2s) *											

Tokak	2	Tokak	C	−	NA	+	+	−	+	S	S
Bereke.54	6	Bereke.54 cold tolerant adapted to southern Kazakstan	C	−	NA	+	−	−	+	S	S
ND-82-10	6	D10	M	−	NA	+	+	−	+	S	S
1-BC-80021	6	Landrace	M	−	NA	+	+	−	+	S	F
Nimrooz	2	Trompillo CMB 74A-432-25B-1Y-1B-1Y-OB	W-S	−	NA	+	+	−	+	S	S
Morex	6	Cree/Bonanza	M	−	NA	+	−	−	−	S	S
